# Hypothyroidism: A Peculiar Presentation

**DOI:** 10.7759/cureus.49819

**Published:** 2023-12-02

**Authors:** Rita V Nogueira, Rui Lima, Carina Parente, Pedro Liberal, Lucia Santos

**Affiliations:** 1 Family Medicine, USF (Unidades de Saúde Familiar) Santa Maria, ACES (Agrupamento de Centros de Saúde) Grande Porto II, Gondomar, PRT

**Keywords:** clinical history, thyroid-stimulating hormone (tsh), levothyroxine, hypothyroidism, myopathy

## Abstract

Hypothyroidism constitutes a prevalent pathology, with up to 80% of afflicted individuals displaying associated muscular symptoms. However, these symptoms rarely appear as the first or only manifestation of hypothyroidism.

We report the case of a previously healthy 21-year-old man, diagnosed with hypothyroid myopathy after experiencing intense myalgias and cramps, which were relieved by oral administration of levothyroxine. This case demonstrates the significance of considering thyroid-related conditions when patients present muscular symptoms as these can represent the only or initial indicator of hypothyroidism. Timely thyroxine supplementation leads to the gradual resolution of most neuromuscular symptoms.

## Introduction

Hypothyroidism is a prevalent medical condition resulting from inadequate production of thyroid hormones or inappropriate hormone action in target tissues [1]. Thyroid dysfunctions, overt or subclinical, are common endocrine disorders affecting 5%-10% of the general population, with hypothyroidism being more common than hyperthyroidism [2]. Approximately 30%-80% of individuals with hypothyroidism may develop muscular symptoms. However, these symptoms rarely present as the initial or sole manifestation of hypothyroidism. In such cases, when no other typical disease symptoms are present, or there is no prior history of thyroid pathology, these individuals are often misdiagnosed with other causes of myopathy [3,4]. Polymyositis is the most common condition confused with hypothyroid myopathy [5].

The cause of muscle symptoms in hypothyroidism remains mostly unknown. Thyroid hormones play a significant role in cellular metabolism, and their deficiency disrupts normal cellular function. Therefore, a proposed pathophysiological mechanism for muscle impairment in hypothyroidism is through alterations in type II fast-twitch muscle fibers, deposition of glycosaminoglycan, diminished contractility of actin-myosin units, reduced activity of myosin ATPase, and decreased ATP turnover in muscle [2,6,7].

Hypothyroid myopathy has many nonspecific symptoms including myalgia, muscle cramps, and fatigue, which worsen with exercise. The severity of myopathy is correlated with the duration and degree of thyroid hormone deficiency [8,9]. The diagnosis of hypothyroid myopathy requires the presence of hypothyroidism, which is confirmed by measuring thyroid-stimulating hormone (TSH) and free T4 serum levels. Elevation of serum creatine kinase (CK) levels, although not specific, is the most common laboratory finding in hypothyroid myopathy [6].

The American Thyroid Association guidelines support that the detection of persistently increased serum concentrations of one or both muscle enzymes, CK and lactate dehydrogenase (LDH), for at least two weeks, justifies the request for serum TSH to confirm or exclude hypothyroidism [2].

In this article, we present a case of hypothyroid myopathy in a young man whose first and only symptoms were muscular and who was successfully treated with levothyroxine.

## Case presentation

We present the case of a 21-year-old male who required emergency care due to recurrent symptoms of myalgia and cramps, primarily in the lower limbs, with approximately two months of progression which worsened over the last week, especially after soccer training sessions. The patient did not report any additional symptoms like fever, anorexia, or weight loss. Moreover, upon reviewing the individual's personal and family medical history, no pertinent information was disclosed, including any previous indicators of a neuromuscular disease. Consumption of medication, dietary supplements, alcohol, and drugs was excluded.

The patient was a student who practiced soccer three times a week, with an official match on weekends. Although he had no difficulty performing his usual daily activities, muscle pain due to exertion significantly impaired his productivity.

During the physical examination conducted in the emergency department, vital signs were within normal limits, and the neurological examination showed no abnormalities. His musculature was well-developed, with no muscle atrophy or fasciculations, presenting normal muscle strength, sensitivity, and reflexes and no gait disturbances.

Laboratory testing revealed elevated serum CK levels (1023 IU/L) with no other abnormalities detected. Exercise myopathy was initially suspected, and the patient was discharged with a recommendation for reevaluation by his primary care physician, if symptoms persisted, for further investigation.

Approximately one week later, due to persistent symptoms, the patient sought primary healthcare, where laboratory tests were requested to rule out possible causes of myopathy, namely markers of inflammation, hydroelectrolytic changes (potassium, sodium, phosphate), and hormonal changes (thyroid function). He was prescribed analgesics and muscle relaxants and was scheduled for a reevaluation in eight days, with consideration of referral to an external neurology consultation for neuromuscular disease assessment. During the reevaluation appointment, the patient still exhibited symptoms, and the laboratory investigation revealed a free T4 level of 0.16 ng/dL (normal range: 0.7-1.8 ng/dL) and a serum TSH level of 134 mU/L (normal range: 0.4-5.5 mU/mL). The anti‑thyroid peroxidase (anti‑TPO) antibody was positive. CK level was 1578 U/L (normal level: <170 U/L). The remaining parameters were essentially normal.

At this moment, the diagnosis of hypothyroid myopathy was established, and the patient immediately received 100 μg/day of levothyroxine, which was subsequently increased to 125 μg/day. Three months after starting supplementation, there was a clinical and laboratory recovery, with a CK level of 186 U/l and serum TSH level of 5.7 μIU/ml.

## Discussion

Muscular symptoms can be the primary or even the initial and sole symptom of hypothyroidism and consist of muscle weakness, cramps, stiffness, and pain and are usually mild, but can be evident, especially in severe untreated hypothyroidism [4].

This case study demonstrates the role of considering thyroid pathology in patients with muscular symptoms, regardless of the presence of other hypothyroidism symptoms or a prior history of hypothyroidism. In this regard, thyroid dysfunction should be considered in the presence of isolated myopathy, even in the absence of typical systemic symptoms of hypothyroidism, to avoid misdiagnosing individuals with other causes of myopathy [10,11]. Salvarani et al. reported two patients with a muscular syndrome in which muscle symptoms combined with a marked elevation in serum muscle enzymes led to a misdiagnosis of polymyositis [3]. However, the laboratory findings revealed highly elevated TSH, and, therefore, they were diagnosed as having hypothyroid myopathy.

In our patient, the diagnosis of hypothyroid myopathy was supported by laboratory tests as well as clinical and laboratory responses to thyroid hormone replacement as mentioned by Salvarani et al., where both patients had few features suggesting hypothyroidism, and only thyroid function tests confirmed the diagnosis [3].

Muscular symptoms are reversible with timely diagnosis and immediate treatment, including appropriate supplementation and restoration of thyroid function. Most symptoms disappear within a year after proper hormone replacement [12-15]. Leonardi et al. relate a case of a rare syndrome due to severe Hashimoto thyroiditis where the administration of levothyroxine eliminated all clinical manifestations of hypothyroidism, including muscle symptoms [12].

The decline of the muscle enzyme levels may occur slowly, varying from weeks, months, or even years from the start of replacement therapy [2], and in our patient's case, CK levels decreased to normal after three months of thyroid hormone replacement as happened in the case of an individual with severe muscle symptoms presented by Lee et al [4].

The clinical variance in the hypothyroidism presentation emphasizes the importance of conducting a global examination of patients presenting with myalgias. In this way, the evaluation of an individual presenting progressive muscle symptoms should begin with a detailed anamnesis and a comprehensive physical examination, including a neurological assessment, followed by laboratory studies to rule out underlying medical disorders, including inflammatory and metabolic processes, like hormonal or electrolyte abnormalities, infections or neoplasias.

Given the extensive literature on hypothyroidism and the elevated prevalence of this pathology in the general population, we consider our case to be a valuable addition to current reports. Its atypical presentation makes it an asset for practitioners seeking a broader understanding of this condition, serving as an alert for a more attentive exploration of muscular symptoms.

## Conclusions

Hypothyroid myopathy is not uncommon in general practice. However, its diagnosis in the absence of other typical hypothyroidism manifestations is not straightforward. Because of this, family doctors should be aware of this rare presentation of primary autoimmune hypothyroidism.

Physicians must remain aware of this less common presentation of hypothyroidism, enabling rapid and accurate diagnosis. Therefore, a comprehensive medical history investigation, an appropriate neuromuscular examination, and the determination of free TSH and T4 serum levels are recommended as initial steps to link myopathy to hypothyroidism and the autoimmune nature of thyroid insufficiency. Further studies, such as thyroid antibody testing and imaging studies, might be necessary for a more precise diagnosis.
